# The Medicine Stamp Question

**Published:** 1902-10-11

**Authors:** 


### THE MEDICINE STAMP QUESTION.

According to the testimony of many chemists it seems probable that the ordinary dispensing of medical prescriptions will before long be a thing of the past. On the one hand the makers of patent medicines so persistently thrust their preparations upon the public that the latter insensibly come to have recourse to them without consulting either doctors or chemists, while on the other, many doctors themselves now prescribe proprietary medicines. Whether this is a desirable state of things may well be questioned. At any rate the modern fashion in drugs is being turned to account by the Inland Revenue authorities in a way which is exciting a great deal of agitation among pharmacists. How far the recent prosecutions were solely dictated by a desire on the part of the Government to stop the modern practice or whether the revenue derivable from the tax on patent medicines is the main consideration are all moot points on which we are perhaps not likely to receive enlightenment.

A test case is about to come before the High Court by way of appeal from a decision of Mr. Dickinson, that ammoniated tincture of quinine B.P. may be sold by chemists with a label recommending its use for influenza and colds without the medicine stamp. The argument for the chemist is in effect that he is not the original or first vendor of the tincture which has been in use for many years, the formula for its preparation being in the British Pharmacopœia and well known. Somerset House contends that the chemist being the person in whose hands the bottle first became chargeable with duty by affixing the label was the first vendor of it, and that as he recommended it by the label the tincture was not within the exemption contained in the Medicine Stamp Act. It will be interesting to note the result of this appeal as it is understood that many other similar preparations will be attacked on the same ground, so that a lively state of warfare between the Government and the chemists may be expected. If the former win it may do something to revive legitimate dispensing, but it is probable that the profits on proprietary articles are sufficient to allow of the tax; and this may be taken into account by the authorities at Somerset House.

